# Cooperative Jamming and Relay Selection for Covert Communications Based on Reinforcement Learning

**DOI:** 10.3390/s25196218

**Published:** 2025-10-07

**Authors:** Jin Qian, Hui Li, Pengcheng Zhu, Aiping Zhou, Shuai Liu, Fengshuan Wang

**Affiliations:** 1 College of Information Engineering, Taizhou University, Taizhou 225300, China; qianjin@tzu.edu.cn (J.Q.); zhupcnt@tzu.edu.cn (P.Z.); zhouaiping@tzu.edu.cn (A.Z.); liushuai@tzu.edu.cn (S.L.); 2 School of Computer Science and Engineering, Xi’an University of Technology, Xi’an 710048, China; fswang@stu.xaut.edu.cn

**Keywords:** covert communications, relay selection, cooperative jamming, covert rate, reinforcement learning

## Abstract

To overcome the obstacles of maintaining covert transmissions in wireless networks employing collaborative wardens, we develop a reinforcement learning framework that jointly optimizes cooperative jamming strategies and relay selection mechanisms. The study focuses on a multi-relay-assisted two-hop network, where potential relays dynamically act as information relays or cooperative jammers to enhance covertness. A reinforcement learning-based relay selection scheme (RLRS) is employed to dynamically select optimal relays for signal forwarding and jamming; the framework simultaneously maximizes covert throughput and guarantees warden detection failure probability, subject to rigorous power budgets. Numerical simulations reveal that the developed reinforcement learning approach outperforms conventional random relay selection (RRS) across multiple performance metrics, achieving (i) higher peak covert transmission rates, (ii) lower outage probabilities, and (iii) superior adaptability to dynamic network parameters including relay density, power allocation variations, and additive white Gaussian noise (AWGN) fluctuations. These findings validate the effectiveness of reinforcement learning in optimizing relay and jammer selection for secure covert communications under colluding warden scenarios.

## 1. Introduction

### 1.1. Background

To counter the growing security threats in contemporary wireless systems, physical layer security (PLS) has become a pivotal area of investigation, offering robust protection against eavesdropping and other malicious activities through inherent channel characteristics [1,2,3]. In contrast to conventional encryption techniques dependent on algorithmic security, PLS techniques such as covert communication focus on hiding the very existence of transmission from malicious eavesdroppers, known as wardens [4]. This paradigm shift is driven by the growing demand for secure communication in sensitive applications ranging from military battlefield networks to Internet of Things (IoT) deployments, where even the detection of communication activity could compromise mission-critical operations or user privacy [5,6,7]. Alongside physical-layer security (PLS) techniques such as cooperative jamming and relay selection, alternative paradigms for covert communication have been explored. Among these, coherent chaos-based systems are notable for their use of deterministic, yet aperiodic and noise-like, chaotic signals as carriers. This inherent property makes the transmitted signal spectrally indistinguishable from background noise, providing a natural layer of covertness against detection by a warden who is unaware of the specific chaotic system used for modulation and demodulation [8]. For instance, military communication systems utilizing distributed sensor networks require robust covertness to avoid detection by colluding wardens, while IoT devices with limited power budgets need energy-efficient solutions that balance communication reliability and covertness [9]. The increasing sophistication of wardens, particularly their ability to collaborate and share detection information, poses significant threats to conventional covert communication strategies, necessitating innovative approaches that can adapt to complex and evolving network conditions [10,11,12,13].

Traditional covert communication methods often rely on static strategies such as fixed power control or random relay selection (RRS), which are fundamentally ill-equipped to maintain performance in dynamic environments with multiple, sophisticated adversaries [14]. For example, RRS schemes completely disregard real-time channel state information and network topology, leading to suboptimal covert rates and high transmission outage probabilities, especially when faced with adaptive wardens [15]. The challenge is amplified by the increasing ability of wardens to collaborate, sharing detection information and using techniques like equal gain combining (EGC) to significantly improve their detection accuracy. These evolving threats expose the urgent need for intelligent, adaptive defense mechanisms that can dynamically optimize network resources to maintain covertness without sacrificing reliability [16,17,18,19]. Multi-agent reinforcement learning (MARL) approaches, particularly the Deep Deterministic Policy Gradient (DDPG) offer particular advantages by enabling distributed decision-making among network nodes [20]. By modeling the interaction between relays and jammers as a Markov game, MARL approaches can dynamically optimize relay selection and jamming strategies to achieve the maximization of the covert transmission rate and maintenance of warden detection error probability above threshold [21,22]. This paper builds on these advancements by proposing a novel RL-based relay selection (RLRS) scheme that integrates cooperative jamming, addressing the limitations of traditional methods and delivering an adaptive defense mechanism against coordinated wardens.

### 1.2. Motivation and Contributions

The primary motivation for this work stems from the critical gap between the capabilities of traditional covert communication schemes and the demands of modern secure networks. Static methods like RRS are proving inadequate against the growing threat of colluding wardens, who leverage collaborative signal processing to enhance their detection capabilities. This escalating threat, particularly in mission-critical military and IoT applications, necessitates a paradigm shift from fixed strategies to adaptive, intelligent systems that can learn and respond to changing network conditions and adversarial behaviors in real-time. The potential of multi-agent reinforcement learning to facilitate decentralized, context-aware decision-making provides a compelling pathway to address these challenges, motivating our development of an integrated relay selection and cooperative jamming framework. This work makes three key advancements, each addressing key limitations in existing covert communication research:

- We introduce an innovative relay selection approach utilizing reinforcement learning principles which dynamically optimizes relay selection for signal forwarding and cooperative jamming, enabling adaptive performance in dynamic networks. Unlike random relay selection, which ignores channel conditions and warden activity, RLRS employs the multi-agent deep reinforcement learning algorithm to make context-aware decisions. This simultaneous optimization of signal quality enhancement and strategic jamming disruption maximizes covert rates while maintaining high detection error probability at colluding wardens.
- We develop a Markov game model to formalize relay–jammer interactions as a multi-objective optimization problem, integrating network state information including channel fading, path loss, and warden collusion intensity. The model’s state space incorporates historical channel data and reflection matrices, while its action space focuses on balancing communication quality and covertness through optimized relay/jammer selection. This framework enables distributed relays to learn optimal strategies via trial-and-error interactions, adapting to varying collusion intensities in (*m*; *M*) scenarios where wardens use equal gain combining for detection.
- Extensive simulations validate RLRS’s superiority over RRS across key metrics: maximum covert rate, transmission outage probability, and robustness to network variations. Results show RLRS steadily improves covert rate with more relays (unlike RRS’s random selection which yields no gains), reduces outage probability through spatial diversity and cooperative jamming (outperforming RRS even under high AWGN), and identifies optimal jamming power levels to avoid self-interference while disrupting detection. These findings confirm RL-based approaches’ effectiveness for covert communications and provide insights for future physical layer security designs.

### 1.3. Related Work

Relay selection focuses on dynamically identifying optimal relay nodes from potential candidates to either forward confidential signals or act as cooperative jammers, aiming to enhance communication quality, maximize covert rate, and reduce transmission outage probability in wireless networks [23,24,25]. Cooperative jamming technology involves deploying friendly jamming nodes to emit artificial interference, which disrupts the detection capabilities of colluding wardens while minimizing interference to legitimate communication links, thereby balancing the trade-off between covertness and transmission reliability [26,27,28]. Reinforcement learning, particularly multi-agent frameworks like MADDPG, enables distributed relays to adaptively optimize relay–jammer selection strategies through trial-and-error interactions with network states (e.g., channel fading, collusion intensity), dynamically improving covert communication performance under dynamic and adversarial environments [29,30,31].

Relay selection enhances wireless network reliability, efficiency, and security, especially in covert/cooperative scenarios requiring adaptive decisions amid dynamic conditions and adversarial threats [23,24,25]. In wireless networks employing multiple relays with jamming-capable wardens, Zhao et al. analyze the achievable covert communication performance [23]. Lyu et al. examine optimal source node selection and resource distribution strategies for dual-hop wireless relay architectures, proposing a credit mechanism-based source selection approach [24]. Mensi et al. investigate the RIS selection strategy in wireless vehicular communications, where vehicles select the RIS offering the highest SNR to optimize communication [25].

Cooperative jamming enables security communications in wireless networks, critical for maintaining covert communication against sophisticated eavesdroppers and colluding wardens [26,27,28]. Hu et al. introduce a low-probability-of-intercept communication method for cognitive radio systems utilizing Poisson-distributed jamming nodes, analyzing detection performance at Willie under PT-Willie collaboration and non-collaboration modes [26]. Dang et al. present a collaborative jamming system for UAV swarms employing wireless power transfer, analyzing SOP and optimizing node placement [27]. Wang et al. develop a federated learning security framework employing collaborative jamming, which reduces communication delays through an incentive-based coordination mechanism [28].

Reinforcement learning addresses dynamic wireless optimization challenges via adaptive decision-making, surpassing static and heuristic approaches [29,30,31]. Wen et al. propose cooperative jamming for RIS-enabled symbiotic radio, optimizing beamforming and reflection coefficients to enhance secrecy performance [29]. Jia et al. comprehensively analyze game-theoretic and reinforcement learning approaches for jamming mitigation in wireless networks, examining both current challenges and potential countermeasures [30]. In Chafii et al., EC-MARL enables autonomous decision-making in 6 G via learned communication protocols for multi-agent cooperative tasks [31].

## 2. System Model

### 2.1. Signal Transmission Model

**Figure 1:**
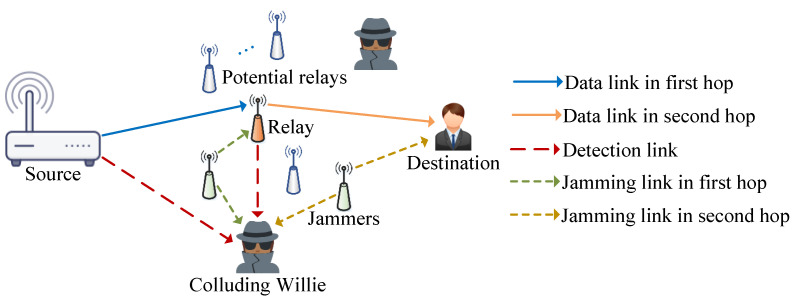
System model.

Depicted in Figure 1, this study investigates a two-hop wireless communication network with multi-relay assistance in the presence of colluding wardens, Willies. The system consists of a source base station (*S*), a destination terminal (*D*), several candidate relays, and multiple colluding wardens, Willies (*W*). With the support of chosen relays, the source base station secretly sends confidential data to the destination, while the Willies strive to identify if the source or any relay is transmitting sensitive information. Each candidate relay can dynamically function as either an information relay or a cooperative jammer based on communication needs, either assisting in the transmission of confidential signals or generating interference to disrupt the Willies, thereby maintaining the communication’s covertness. The source base station and information relays both transmit confidential data using power $P_{s}$, whereas cooperative jamming nodes emit jamming signals with power $P_{j}$. Here, $P_{s}$ and $P_{j}$ are adhering to peak transmission power limitations $P_{\max}$. It is assumed that all entities, including the source base station, relays, and jammers are outfitted with single antennas.

We analyze an (*m*; *M*) warden collaboration scenario where any subset of *m* wardens ($W_{1},W_{2},\cdot \cdot \cdot ,W_{m}$, with 1≤m≤M) are capable of merging their collected observations to identify the transmission. Here, *m* represents the collaboration strength, quantifying the degree of warden cooperation. Among *m* cooperating wardens, a designated super warden $W_{T}$ aggregates individual received signal energy measurements from all members. Our analysis assumes each warden possesses only statistical CSI for its link from source *S*, without instantaneous CSI knowledge. Consequently, the EGC method is adopted in $W_{T}$ for collusive detection, aggregating the signal energy measurements from all *m* collaborating nodes to identify potential transmissions from Alice. This approach proves particularly advantageous in statistical CSI scenarios, as it eliminates the need for instantaneous channel knowledge while maintaining low computational overhead. The EGC technique has become prevalent in modern detection systems due to these operational benefits, particularly in covert communication applications where channel information is limited. The collaboration framework offers significant practical utility by dynamically modeling different cooperation intensities among surveillance nodes. This approach proves particularly valuable in cellular networks where geographically distributed base stations monitor for illicit transmissions. Upon detecting suspicious activity in a localized area, the system can selectively engage a subset of detectors (e.g., *m* of *M* available nodes) for coordinated analysis, while inactive stations continue serving authorized users without interruption maintaining both surveillance effectiveness and network QoS.

It is important to clarify the scope of security provided by this system. Our work focuses on PLS, where the primary objective is to achieve covertness that is, to hide the very existence of a transmission from a warden. This approach is designed to function as a foundational security layer, complementary to traditional cryptographic methods. We assume that the confidential data transmitted by the source is already encrypted using upper-layer cryptographic algorithms. Therefore, the goal of our RLRS scheme is not to protect the message content from being deciphered, but to prevent a warden from even detecting that a communication is taking place. This layered security strategy ensures that even in the unlikely event a transmission is detected, the message content remains secure against interception and brute-force attacks.

It is important to clarify the scope of the threat model considered in this paper. Our work focuses specifically on countering a sophisticated and highly effective passive attack: detection by a network of colluding wardens employing energy detection with Equal Gain Combining (EGC). This scenario represents a significant threat in practical systems, as collaboration drastically improves the warden network’s ability to detect faint signals. While other types of attacks exist (e.g., active jamming, pilot spoofing), our objective is to design a robust defense against this specific, challenging detection strategy, which serves as a critical benchmark for covert communication systems.

### 2.2. Channel Model

The wireless channels exhibit quasi-static fading with statistically independent behavior, incorporating path loss that varies according to propagation distance with exponent α. Concerning fading properties, all channel coefficients stay unaltered within each time slot while demonstrating statistically independent behavior across consecutive slots.

The wireless channels between all node pairs *S* and *R*, *S* and $W_{g}$, *R* and *D*, *R* and $W_{g}$, any friendly jammer ($J_{i}$) and $W_{g}$, $J_{i}$ and *R*, as well as $J_{i}$ and *D*, exhibit quasi-static Rayleigh fading, modeled as independent and identically distributed complex Gaussian random variables CN(0,1), denoted by $h_{s,r}$, $h_{s,w_{g}}$, $h_{r,d}$, $h_{r,w_{g}}$, $h_{j_{i},w_{g}}$, $h_{j_{i},r}$, $h_{j_{i},d}$ respectively. Similarly, the spatial configuration of all nodes is characterized by the following distances $d_{s,r}$, $d_{s,w_{g}}$, $d_{r,d}$, $d_{r,w_{g}}$, $d_{j_{i},w_{g}}$, $d_{j_{i},r}$, $d_{j_{i},d}$ in sequence. $|h_{i,j}{|}^{2}$ represents the respective channel gain where $ij\in \{sr,sw_{g},rd,rw_{g},j_{i}w_{g},j_{i}r,j_{i}d\}$. The channel gain model accounts for the antenna gain of transceiving antennas and the spatial separation between any pair of nodes. Channel noise is modeled using AWGN with variance $\sigma_{a}^{2}$, where $\sigma_{a}^{2}∼\mathrm{CN}\left(0,\sigma_{a}^{2}\right)$ for $a\in \{r,d,w_{g}\}$. The noise variance is given by $\sigma_{a}^{2}=2N_{02}B$, where *B* denotes channel bandwidth. All wireless links experience independent Rayleigh-distributed fading.

When *S* sends a confidential signal, the signals received by *R* and Willie $w_{g}$ (where g∈m+1,m+2,…,M) are expressed as

(1) $$\begin{array}{cc} y_{r} & =\sqrt{P_{s}}h_{s,r}x_{s}+\sum_{j_{i}}\sqrt{P_{j}}h_{j_{i},r}x_{j}+\sigma_{r}^{2}, \end{array}$$

(2) $$\begin{array}{cc} y_{w_{g}}^{f} & =\sqrt{P_{s}}h_{s,w_{g}}x_{s}+\sum_{j_{i}}\sqrt{P_{j}}h_{j_{i},w_{g}}x_{j}+\sigma_{w_{g}}^{2}, \end{array}$$

where $x_{s}$ denotes the signal sent by *S*, $x_{j}$ represents the transmission signal from jammers, and $\sigma_{r}^{2}$ and $\sigma_{w_{g}}^{2}$ stand for the AWGN at *R* and Willie, respectively.

Similarly, when *R* forwards the confidential signal, the signals received at *D* and by Willie $w_{g}$ (where g∈m+1,m+2,…,M) are given by

(3) $$\begin{array}{cc} y_{d} & =\sqrt{P_{s}}h_{r,d}x_{r}+\sum_{j_{i}}\sqrt{P_{j}}h_{j_{i},d}x_{j}+\sigma_{d}^{2}, \end{array}$$

(4) $$\begin{array}{cc} y_{w_{g}}^{s} & =\sqrt{P_{s}}h_{r,w_{g}}x_{r}+\sum_{j_{i}}\sqrt{P_{j}}h_{j_{i},w_{g}}x_{j}+\sigma_{w_{g}}^{2}, \end{array}$$

where $x_{r}$ refers to the signal emitted by *R*, $x_{j}$ stands for the signal sent by jammers, and $\sigma_{d}^{2}$ and $\sigma_{w_{g}}^{2}$ denote the AWGN at *D* and Willie, respectively.

Within the (*m*; *M*) collusion setting involving *m* colluding wardens, employing EGC, the super warden $W_{T}$ processes incoming signals during the first and second hops, denoted as $y_{w_{t}}$, is given by

(5) $$\begin{array}{c} y_{w_{t}}^{f}=\sum_{n=1}^{m}\left(\sqrt{P_{s}}h_{s,w_{t}}x_{s}+\sum_{j_{i}}\sqrt{P_{j}}h_{j_{i},w_{t}}x_{j}+\sigma_{w_{t}}^{2}\right) \end{array}$$

(6) $$\begin{array}{c} y_{w_{t}}^{s}=\sum_{n=1}^{m}\left(\sqrt{P_{s}}h_{r,w_{t}}x_{r}+\sum_{j_{i}}\sqrt{P_{j}}h_{j_{i},w_{t}}x_{j}+\sigma_{w_{t}}^{2}\right) \end{array}$$

The instantaneous SINR at both the relay *R* and destination *D* nodes can be formulated as

(7) $$\begin{array}{c} {\mathrm{SINR}}_{r}=\frac{P_{s}{\left|h_{s,r}\right|}^{2}}{\sum_{j_{i}}P_{j}{\left|h_{j_{i},r}\right|}^{2}+\delta_{r}^{2}}, \end{array}$$

(8) $$\begin{array}{c} {\mathrm{SINR}}_{d}=\frac{P_{s}{\left|h_{r,d}\right|}^{2}}{\sum_{j_{i}}P_{j}{\left|h_{j_{i},d}\right|}^{2}+\delta_{d}^{2}}. \end{array}$$

### 2.3. Relay Selection Scheme

(1) RRS: In this approach, *S* randomly picks a candidate from all possible relays to aid in transmitting the message to destination terminal *D*.

(2) RLRS: Within this framework, we introduce a relay selection mechanism grounded in RL, termed RLRS, to facilitate the covert delivery of sensitive data. By utilizing RL techniques, the system dynamically chooses suitable relay nodes for information transfer from among available candidates, forwarding signals originating from the source to the intended destination. Concurrently, it pinpoints appropriate cooperative jamming nodes to generate jamming signals designed to disrupt Willies throughout transmission. This integrated strategy of relay and jammer selection guarantees the covert transmission of private data.

### 2.4. Performance Metrics

Willie performs binary hypothesis evaluating to detect potential covert transmissions between *S* and *D*, where $H_{0}$: Absence of covert communication; $H_{1}$: Active covert transmission. Three key performance metrics are analyzed for assessing covert communication effectiveness: *detection error probability*, transmission outage probability, and covert rate.

(1) Detection error probability: This metric quantifies Willie’s likelihood of making erroneous detection decisions regarding covert transmission presence, formulated as:

(9) $$\begin{array}{c} \xi =P_{\mathrm{FA}}+P_{\mathrm{MD}}. \end{array}$$

Here, ξ denotes probability of detection error. $P_{\mathrm{FA}}$ stands for false alarm rate, that is, the scenario where Willie decides $H_{1}$ holds when $H_{0}$ is actually true, while $P_{\mathrm{MD}}$ indicates the missed detection probability: specifically, when $H_{1}$ is true, Willie judges $H_{0}$ to be the case.

(2) Transmission outage probability: A communication outage occurs if the received signal power at either *R* or *D* falls beneath the minimum sensitivity threshold θ, preventing successful message decoding.

(3) Covert rate: covert rate represents the achievable transmission rate from *S* to *D* while ensuring Willie’s detection error probability remains above a specified threshold.

## 3. Covert Performance Under RRS Scheme

### 3.1. Detection at Colluding Willies

Based on these assumptions, the colluding Willies’ hypothesis evaluating process is initially examined for the first hop, with an identical approach applied to the second hop, enabling subsequent derivation of the detection error probability. For the RRS approach, the signal $y_{W_{t}}$ observed by the super warden $W_{t}$ during the initial transmission phase can be expressed as

(10) $$\begin{array}{cc} H_{0}:y_{w_{t}}\left(t\right) & =\sum_{j_{i}}\sqrt{P_{j}}h_{j_{i},w_{t}}x_{j}+\sigma_{w_{t}}^{2}, \end{array}$$

(11) $$\begin{array}{cc} H_{1}:y_{w_{t}}\left(t\right) & =\sqrt{P_{s}}h_{s,w_{t}}x_{k}+\sum_{j_{i}}\sqrt{P_{j}}h_{j_{i},w_{t}}x_{j}+\sigma_{w_{t}}^{2}. \end{array}$$

Utilizing radiometer detection, the CBS predetermines a threshold value Γ, based on which the subsequent decision is made.

(12) $$\begin{array}{c} Y_{w_{t}}=\frac{1}{N_{02}}\int_{0}^{\tau }{\left|y_{w_{t}}\left(t\right)\right|}^{2}dt\overset{H_{1}}{\underset{H_{0}}{≷}}\Gamma . \end{array}$$

Here, $Y_{w_{g}}$ denotes the signal energy received by Willie. From Willie’s perspective, it is assumed that the received powers from *S* and the jammers are constant, given by ${\hat{P}}_{s}=\mathrm{Tr}\left(W_{s}H_{s,w_{t}}\right)$ and ${\hat{P}}_{j}=\mathrm{Tr}\left(W_{j}H_{j,w_{t}}\right)$, respectively. Consequently, the energy observed from the primary and jamming transmissions over the duration τ are ${\hat{P}}_{s}\tau$ and ${\hat{P}}_{j}\tau$, respectively.

Under hypothesis $H_{0}$, the received energy $Y_{w_{t}}$ can be characterized by a non-central chi-square distribution featuring 2τB degrees of freedom and non-centrality parameter $\frac{({\hat{P}}_{j})\tau }{N_{02}}$. In contrast, under hypothesis $H_{1}$, $Y_{w_{t}}$ is governed by $\delta =\frac{({\hat{P}}_{s}+{\hat{P}}_{j})\tau }{N_{02}}$. When the value of 2τB becomes sufficiently large, the central limit theorem (CLT) enables us to approximation of underlying distribution of $Yw_{t}$ under both hypotheses as follows:

(13) $$\begin{array}{c} Y_{w_{t}}|H_{0}∼N\left(2\tau B\;+\;\frac{{\hat{P}}_{j}\tau }{N_{02}},4\tau B\;+\;\frac{4{\hat{P}}_{j}\tau }{N_{02}}\right), \end{array}$$

(14) $$\begin{array}{c} Y_{w_{t}}|H_{1}∼N(2\tau B+\frac{({\hat{P}}_{s}+{\hat{P}}_{j})\tau }{N_{02}},4\tau B\\ \;\;\;\;\;\;\;+\frac{4({\hat{P}}_{s}+{\hat{P}}_{j})\tau }{N_{02}}). \end{array}$$

Here, $N(\mu ,\delta^{2})$ denotes a normal distribution.

Willie determines whether source *S* has transmitted a message based on the power of the signal it receives. In this study, we characterize $P_{\mathrm{FA}}$ as the false alarm probability, corresponding to Willie incorrectly concluding $H_{1}$ while $H_{0}$ holds true. Conversely, $P_{\mathrm{MD}}$ denotes missed detection likelihood, where Willie accepts $H_{0}$ despite $H_{1}$ being the true hypothesis. Leveraging the approximations in (14), the corresponding expressions for $P_{\mathrm{FA}}$ and $P_{\mathrm{MD}}$ can be derived as shown in Equations (15) and (16):

(15) $$\begin{array}{cc} P_{\mathrm{FA}} & =\mathrm{prob}\left(Y_{w_{g}}>\Gamma |H_{0}\right)=\left\{\begin{array}{cc} 1, & \Gamma \leq \delta_{w_{g}}^{2}\\ Q\left(\begin{array}{c} \frac{\Gamma -(2B\tau +\frac{{\hat{P}}_{j}\tau }{N_{02}})}{\sqrt{4B\tau +\frac{4{\hat{P}}_{j}\tau }{N_{02}}}} \end{array}\right), & \Gamma >\delta_{w_{g}}^{2} \end{array}\right. \end{array}$$

(16) $$\begin{array}{cc} P_{\mathrm{MD}} & =\mathrm{prob}\left(Y_{w_{g}}<\Gamma |H_{1}\right)=\left\{\begin{array}{cc} 0, & \Gamma \leq {\hat{P}}_{j}\tau +\delta_{w_{g}}^{2}\\ 1-Q\left(\begin{array}{c} \frac{\Gamma -(2B\tau +\frac{({\hat{P}}_{s}+{\hat{P}}_{j})\tau }{N_{02}})}{\sqrt{4B\tau +\frac{4({\hat{P}}_{s}+{\hat{P}}_{j})\tau }{N_{02}}}} \end{array}\right), & \Gamma >{\hat{P}}_{j}\tau +\delta_{w_{g}}^{2} \end{array}\right. \end{array}$$

Here, Q(·) represents the tail distribution function of a standard normal random variable, expressed as:

(17) $$\begin{array}{c} Q\left(t\right)=\frac{1}{\sqrt{2\pi }}\int_{t}^{+\infty }\exp\left(\frac{-x^{2}}{2}\right)dx.\; \end{array}$$

Since false alarm and missed detection represent two distinct types of detection errors by the warden, the covert communication performance is evaluated through the detection error probability (DEP) metric, denoted ξ in (18). Transmission from *S* is deemed covert when the condition ξ≥1−ϵ is satisfied, where ϵ represents the predefined covertness threshold.

(18) $$\begin{array}{cc} \xi =P_{\mathrm{FA}}+P_{\mathrm{MD}}= & \left\{\begin{array}{cc} 1, & \Gamma \leq \delta_{w_{g}}^{2}\\ Q\left(\frac{\Gamma -(2B\tau +\frac{{\hat{P}}_{j}\tau }{N_{02}})}{\sqrt{4B\tau +\frac{4{\hat{P}}_{j}\tau }{N_{02}}}}\right), & \delta_{c}^{2}<\Gamma \leq {\hat{P}}_{j}\tau +\delta_{c}^{2}\\ 1-Q\left(\frac{\Gamma -(2B\tau +\frac{({\hat{P}}_{s}+{\hat{P}}_{j})\tau }{N_{02}})}{\sqrt{4B\tau +\frac{4({\hat{P}}_{s}+{\hat{P}}_{j})\tau }{N_{02}}}}\right)+Q\left(\frac{\Gamma -(2B\tau +\frac{{\hat{P}}_{j}\tau }{N_{02}})}{\sqrt{4B\tau +\frac{4{\hat{P}}_{j}\tau }{N_{02}}}}\right), & \Gamma >{\hat{P}}_{j}\tau +\delta_{c}^{2} \end{array}\right. \end{array}$$

### 3.2. Covert Rate Modeling

Effective modeling and analysis of the fundamental covert rate require initially establishing the transmission outage probability for the *S* to *D* link. The derivation of this probability is presented in the subsequent theorem.

**Theorem** **1.** *Represented transmission outage probability as $P_{\mathrm{out}}$, it takes the form*

(19) $$\begin{array}{c} P_{\mathrm{out}}=1-\exp\left(-\frac{\lambda (\sigma_{r}^{2}+\sigma_{d}^{2})}{P_{s}}\right){\left[\frac{P_{s}}{\lambda P_{j}+P_{s}}\right]}^{2}. \end{array}$$

**Proof.**  In the analyzed two hop wireless network, successful transmission occurs only when both *S* to *R* and *R* to *D* links operate without outage. Consequently, the overall outage probability $P_{\mathrm{out}}$ is given by a sequential manner: We first define the overall outage probability as the union of two independent events, i.e., the SINR falling below the threshold at either the relay or the destination. Next, we provide the equivalent complementary probability expression that involves both SINR terms exceeding the threshold. Finally, we show the independence property explicitly and derive the closed-form expression step by step, as follows:

(20) $$\begin{array}{cc} P_{\mathrm{out}} & =P\left({\mathrm{SINR}}_{r}<\lambda ⋃{\mathrm{SINR}}_{d}<\lambda \right), \end{array}$$

(21) $$\begin{array}{cc} \; & =1-P\left({\mathrm{SINR}}_{r}\geq \lambda ⋂{\mathrm{SINR}}_{d}\geq \lambda \right), \end{array}$$

(22) $$\begin{array}{cc} \; & =1-P\left({\mathrm{SINR}}_{r}\geq \lambda \right)P\left({\mathrm{SINR}}_{d}\geq \lambda \right), \end{array}$$

where the received SINR at relay *R* (SINR_*r*_) and destination *D* (SINR_*d*_) follow Equations (7) and (8), respectively.

The channel gain statistics follow distinct distributions: $\sum_{j_{i}}{\left|h_{j_{i},r}\right|}^{2}$ and $|\sum_{j_{i}}h_{j_{i},d}{|}^{2}$ adhering to

(23) $$\begin{array}{c} f_{|h_{j,r}{|}^{2}}\left(x\right)=f_{|h_{j,d}{|}^{2}}\left(x\right)=e^{-x},0\leq x\leq \infty , \end{array}$$

then,

(24) $$\begin{array}{cc} P({\mathrm{SINR}}_{r}\geq \lambda ) & =P\left[|h_{s,r}{|}^{2}\geq \frac{\lambda (P_{j}|h_{j,r}{|}^{2}+\sigma_{r}^{2})}{P_{s}}\right]\\ \; & =E\left[\exp\left(-\frac{\lambda (P_{j}|h_{j_{i},r}{|}^{2}+\sigma_{r}^{2})}{P_{s}}\right)\right]\\ \; & =\exp\left(\frac{-\lambda \sigma_{r}^{2}}{P_{s}}\right)E\left[\exp\left(\frac{-\lambda |h_{j,r}{|}^{2}P_{j}}{P_{s}}\right)\right]\\ \; & =\exp\left(-\frac{\lambda \sigma_{r}^{2}}{P_{s}}\right)\left[\frac{P_{s}}{\lambda P_{j}+P_{s}}\right]. \end{array}$$

Similarly

(25) $$\begin{array}{c} P\left({\mathrm{SINR}}_{d}\geq \lambda \right)=\exp\left(-\frac{\lambda \sigma_{d}^{2}}{P_{s}}\right)\left[\frac{P_{s}}{\lambda P_{j}+P_{s}}\right]. \end{array}$$

By combining the results from Equations (24) and (25) with Equation (19), a closed-form solution for $P_{\mathrm{out}}$ can be derived. □

The achievable covert transmission rate $R_{s,d}$ for the *S*-to-*D* link is formulated using previously derived outage probability $P_{\mathrm{out}}$, and can be represented mathematically as

(26) $$\begin{array}{c} R_{s,d}=\left(1-P_{\mathrm{out}}\right)\min\left\{R_{s,r},R_{r,d}\right\}. \end{array}$$

Here, the achievable covert transmission rate from source *S* to relay *R* is represented as $R_{s,r}=\log_{2}\left(1+{\mathrm{SINR}}_{r}\right)$, while the covert rate from relay *R* to destination *D* is given by $R_{r,d}=\log_{2}\left(1+{\mathrm{SINR}}_{d}\right)$, respectively.

### 3.3. Covert Rate Optimization

Building upon these findings, we observe that enhanced covert communication reliability correlates strongly with higher transmission rates. This motivates our formulation of an optimization framework aimed at maximizing $R_{s,d}$, subject to the critical constraint of preserving Willie’s detection uncertainty. The resulting covert rate maximization problem is formally expressed as

(27a) $$\begin{array}{cc} \max_{w_{s},w_{j}} & R_{s,d}\; \end{array}$$

(27b) $$\begin{array}{cc} \;s.t. & \;\xi \geq 1-\epsilon , \end{array}$$

(27c) $$\begin{array}{cc} & \;P_{s}\leq P_{\max}, \end{array}$$

(27d) $$\begin{array}{cc} & \;P_{j}\leq P_{\max} \end{array}$$

where ϵ represents the required covertness threshold, while constraints (28c) and (28d) specify the peak transmission power limits for source *S* and all cooperative jammers, respectively.

## 4. Covert Performance Under RLRS Scheme

In this section, we propose a relay selection scheme based on reinforcement learning. Specifically, deep reinforcement learning algorithms are employed to intelligently select appropriate signal relay nodes and cooperative interference nodes from a set of potential relays. Additionally, the transmission power of both the signal source and the cooperative interference nodes is jointly optimized to maximize the covert transmission rate of the wireless communication network.

### 4.1. Covert Rate Optimization

To enhance covert communication reliability through increased transmission rates, we formulate an optimization problem that maximizes $R_{s,d}^{\prime }$ while preserving Willie’s detection uncertainty, building upon established theoretical foundations. The optimization problem that follows is an expression for the covert rate maximization issue:

(28a) $$\begin{array}{cc} \max_{w_{s},w_{j}} & R_{s,d}^{\prime }\; \end{array}$$

(28b) $$\begin{array}{cc} \;s.t. & \;\xi \geq 1-\epsilon , \end{array}$$

(28c) $$\begin{array}{cc} & \;P_{s}\leq P_{\max}, \end{array}$$

(28d) $$\begin{array}{cc} & \;P_{j}\leq P_{\max} \end{array}$$

where the peak transmit power at both source node *S* and all jamming nodes cannot exceed threshold (28c) and the covertness requirement is ϵ.

### 4.2. Multiobjective Optimization Based on Markov Game

For a multi-objective optimizing operation, we create a Markov game. In a five-tuple Markov game, the optimization challenge for beamforming and relay selection is first expressed as = {I,S,A,T,R}. It shows I for the group of agents, S for the group of states, A for the action group, T for the state transfer probability, and R for the reward value. Relays are the intelligent agents in the environment we consider to be a communication network in the presence of colluding Willie. Here are the specifics:

(1) *State space:* The state space $s_{t}$ contains the environmental data which the *R* has observed, encompassing received signal attributes, past channel state information.

(2) *Action space:* For potential relay nodes, the action space space $a_{t}$ includes selecting roles as either signal relays or cooperative interference nodes. The signal relay nodes aim to enhance signal quality at destination, while collaborative disruption nodes are responsible for disrupting Willie’s detection capability, thereby improving the overall covert communication performance of system.

(3) *State Transfer Probability:* The probability of moving transitioning from the current state $s_{t}$ to the subsequent $s_{t+1}$ whenever action *a* is adopted during temporal window *t*. All $s_{t}\in S$ and $a_{t}\in A$ satisfy the following conditions:

(29) $$\begin{array}{cc} T(s_{t+1}|s_{t},a_{t}) & >0, \end{array}$$

(30) $$\begin{array}{cc} \sum_{s_{t+1}\in S}T\left(s_{t+1}|s_{t},a_{t}\right) & =1. \end{array}$$

(4) *Reward:* Wireless communication networks enhance system concealment rates by strategically selecting signal relay nodes and cooperative interference nodes, while jointly optimizing transmission power of both the source and the interference nodes. As a result, the intelligent agent’s instantaneous reward value is depicted as

(31) $$\begin{array}{cc} r_{r_{i}}\left(t\right) & =R_{s,d}^{\prime }. \end{array}$$

### 4.3. Beamforming Optimization and Relay Selection Based on MADDPG

To solve the optimization problem $R_{s,d}^{\prime }$ involving beamforming and relay selection, we propose a multi-agent reinforcement learning strategy based on the MADDPG framework. This approach adopts centralized training alongside decentralized execution. Each agent is equipped with an actor network $\mu_{i}\left(s_{t}^{i}\right)$, a corresponding target actor network $\mu_{i}^{\prime }\left(s_{t+1}^{i}\right)$, a critic network $Qi(s_{t},a_{t})$, and a target critic network $Qi^{\prime }\left(st+1,at+1\right)$. In the MADDPG paradigm, agents are capable of accounting for the behavior of others during decision-making. While actor networks rely solely on local observations, the critic networks are enhanced with global state–action information to guide learning more effectively, see Algorithm 1.

We define $\pi_{i}$ as the policy adopted by agent *i* under the MADDPG framework. The evaluation networks are parameterized by $\theta_{i}^{\mu }$ for the actor and $\theta_{i}^{Q}$ for the critic, both of which are iteratively updated to approach the optimal policy. During training, the interaction between agents and the environment generates encounter tuple $\left(s_{t},a_{t},r_{t},s_{t+1}\right)$, those that are kept in the D. Mini-batches in size ϵ are taken at D randomly to update the evaluation networks. The critic network updates its parameters $\theta_{i}^{Q}$ by minimizing a loss function, which is expressed as follows:

(32) $$\begin{array}{cc} L(\theta_{i}^{Q}) & =E[{\left(Q_{i}\left(s_{t},a_{t}^{i},a_{t}^{-i}|\theta_{i}^{Q}\right)-y_{t}^{i}\right)}^{2}], \end{array}$$

(33) $$\begin{array}{cc} y_{t}^{i} & =r_{t}^{i}+\gamma Q_{i}^{\prime }\left(s_{t+1},a_{t+1}^{i},a_{t+1}^{-i}|\theta_{i}^{Q}\right). \end{array}$$

Here, $Q_{i}^{\prime }\left(\cdot \right)$ denotes target critic network’s state–action worth function. To update actor network, The actor’s policy parameters $\theta_{i}^{\mu }$ are optimized to maximize the objective function. The policy objective function is formulated as follows:

(34) $$\begin{array}{c} J\left(\theta_{i}^{\mu }\right)=E\left[\left(Q_{i}\left(s_{t}^{i},a^{i}|a^{i}\right)=\mu_{i}\left(s_{t}^{i}\right)\right)\right]. \end{array}$$

Here, $\mu_{i}\left(\cdot \right)$ represents the actor evaluation network function, which maps states to actions under the deterministic policy $\pi_{i}$. Instead of directly copying the evaluation network parameters $\theta_{i}^{\mu }$ and $\theta_{i}^{Q}$ to the corresponding target networks, the parameters of target actor $\mu_{i}^{\prime }$ and target critic $Q_{i}^{\prime }$ are adjusted gradually using a soft update mechanism:

(35) $$\begin{array}{cc} \theta_{i}^{\mu^{\prime }} & =\lambda_{a}\theta_{i}^{\mu }+\left(1-\lambda_{a}\right)\theta_{i}^{\mu^{\prime }}, \end{array}$$

(36) $$\begin{array}{cc} \theta_{i}^{Q^{\prime }} & =\lambda_{b}\theta_{i}^{Q}+\left(1-\lambda_{b}\right)\theta_{i}^{Q^{\prime }}, \end{array}$$

where $\lambda_{a}\ll 1$, $\lambda_{b}\ll 1$.
**Algorithm 1** MADDPG algorithm.1:Initialize the evaluation parameters for the actor and critic networks as $\theta_{i}^{\mu }$ and $\theta_{i}^{Q}$, respectively.2:Construct the experience replay buffer D and define a small batch size τ such that τ≪D. Set the action noise as H, the total number of training epochs as E, and the number of training steps per epoch as M.3:**for** each epoch from 1 to E **do**4:   Initialize the environment with a random state $s_{t}^{i}$ for each agent;5:   **for** step *t* = 1 to M **do**6:      Based on the current deterministic policy, each agent selects an action according to $a_{t}^{i}=\mu_{i}\left(s_{t}^{i}\right)+H_{t}$;7:      Execute the selected action $a_{t}^{i}$ and receive the corresponding reward $r_{t}^{i}$;8:      $s_{t}^{i}\leftarrow s_{t+1}^{i}$9:      Store the transition tuple $\left(s_{t}^{i},a_{t}^{i},r_{t}^{i},s_{t+1}^{i}\right)$ in the experience buffer D;10:      **for** each agent **do**11:         Randomly select a subset of experiences $\left(s_{t}^{i},a_{t}^{i},r_{t}^{i},s_{t+1}^{i}\right)$ from D;12:         Define target value as $y_{t}^{i}=r_{t}^{i}+\gamma ;Q_{i}^{\prime }\left(s_{t+1},a_{t+1}^{i},a_{t+1}^{-i}|\theta_{i}^{Q}\right)$;13:         The critic network is optimized through gradient descent on the loss function $L\left(\theta_{i}^{Q}\right)=E\left[{\left(Q_{i}\left(s_{t},a_{t}^{i},a_{t}^{-i}|\theta_{i}^{Q}\right)-y_{t}^{i}\right)}^{2}\right]$;14:         To update the actor network, maximize the policy objective function: $J\left(\theta_{i}^{\mu }\right)=E\left[\left(Q_{i}\left(s_{t}^{i},a^{i}|a^{i}\right)=\mu_{i}\left(s_{t}^{i}\right)\right)\right]$;15:      **end for**16:      Finally, update the parameters of each agent’s target networks using soft updates as follows: $\theta_{i}^{\mu^{\prime }}=\lambda_{a}\theta_{i}^{\mu }+\left(1-\lambda_{a}\right)\theta_{i}^{\mu^{\prime }},\theta_{i}^{Q^{\prime }}=\lambda_{c}\theta_{i}^{Q}+\left(1-\lambda_{c}\right)\theta_{i}^{Q^{\prime }}$.17:   **end for**18: **end for**

## 5. Simulation Results

### 5.1. Simulation Setup

In this section, we detail the simulation environment and parameters used to evaluate the performance of our proposed RLRS scheme and compare it with the baseline RRS method. The network model consists of a source, a destination, multiple colluding wardens, and a set of potential relays distributed randomly in a defined area. To ensure statistical validity and mitigate the effects of random channel variations, all presented results are averaged over 10,000 independent Monte Carlo simulation runs. The key parameters used throughout the simulations are summarized in Table 1, unless explicitly stated otherwise in the analysis of a specific figure.

### 5.2. Performance Analysis of the Proposed RLRS Scheme

**Figure 2:**
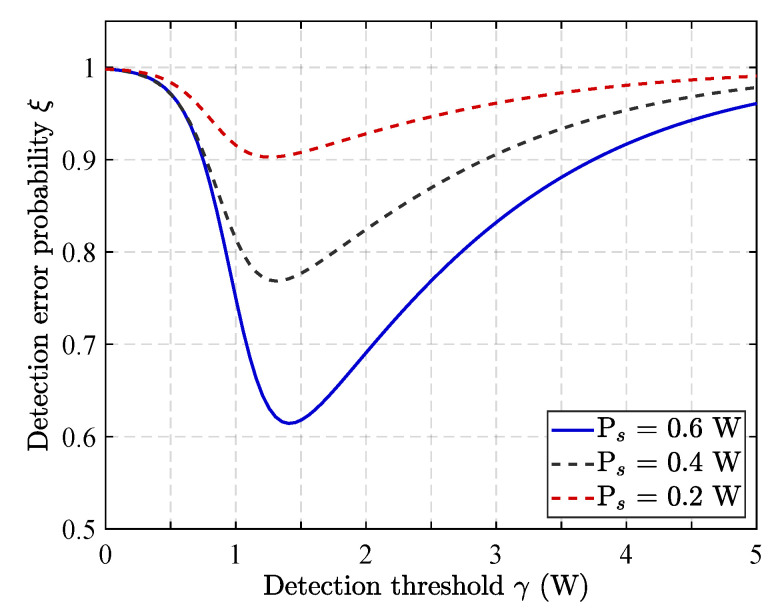
Impact of detection threshold on detection error probability.

Figure 2 shows how Willie’s detection threshold affects the likelihood of a detection error under varying transmit power levels of the source base station in the RLRS scheme. As observed, the detection error probability exhibits a convex relationship with respect to the detection threshold, first declining before reaching a minimum and subsequently increasing. This behavior arises because detection error probability ξ consists of $P_{\mathrm{FA}}$ and $P_{\mathrm{MD}}$. When threshold is low, false alarm probability dominates; thus, increasing threshold reduces overall error. However, as the threshold continues to increase, the miss detection probability becomes dominant, causing the overall error to rise. Additionally, under the same detection threshold, higher transmission power from the source base station leads to a lower detection error probability in Willie. This occurs since higher transmit power reduces the uncertainty at Willie’s receiver, facilitating more reliable detection of ongoing transmissions, thereby reducing his detection uncertainty.

**Figure 3:**
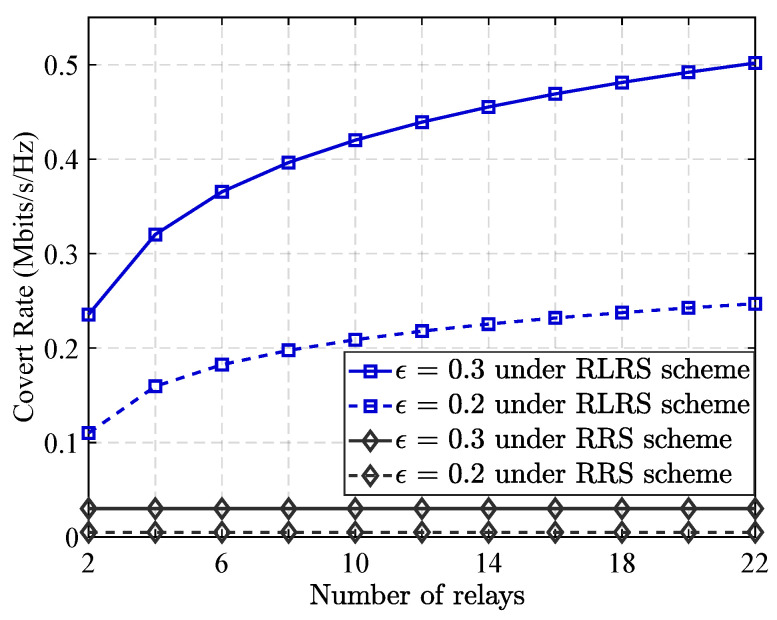
Impact of the number of relays on the maximum covert rate.

Figure 3 demonstrates how relay node quantity impacts covert transmission rates across varying stealth conditions, contrasting the RLRS approach with RRS. As shown, covert communication rate under RLRS method steadily increases as the number of relay nodes grows. This positive trend can be attributed to the reinforcement learning algorithm’s ability to intelligently and dynamically select the most advantageous relay nodes for two key functions: signal forwarding and cooperative jamming. By doing so, the network increase quality and efficiency of confidential information transmission as well as enhances its ability to disrupt the detection capabilities of the warden (Willie). This intelligent relay selection process takes full advantage of the expanded relay pool, allowing the system to exploit more favorable transmission paths and interference opportunities to bolster covert performance. In contrast, the RRS scheme shows no significant improvement in covert communication rate when there are more relay nodes. This is because relay selection under RRS is performed randomly and independently of the network state or strategic considerations. As a result, adding more relay nodes does not reduce the probability of transmission interruption, nor does it contribute to improved concealment from the warden. This highlights the inefficiency of random selection in leveraging network diversity for covert communication purposes.

**Figure 4:**
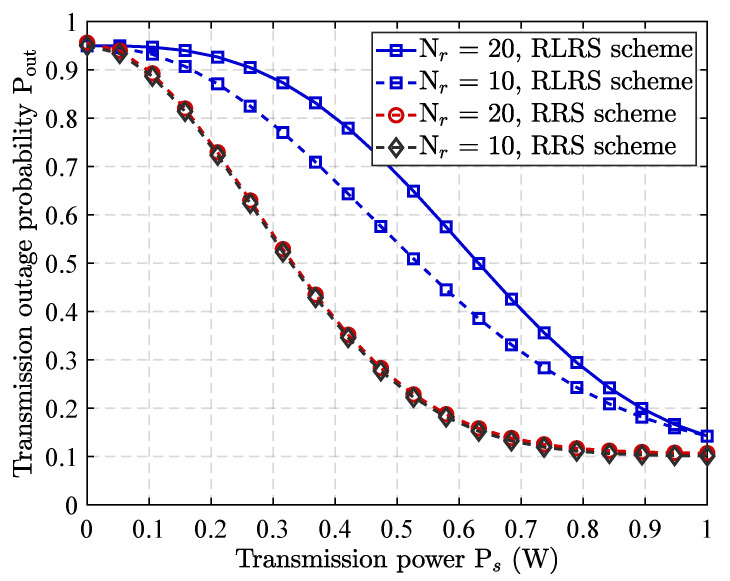
Impact of $P_{s}$ on transmission outage probability under different numbers $N_{r}$ of relay and relay selection scheme.

The Figure 4 depicts the impact of the source base station’s transmit power on the probability of communication interruption, evaluated under varying numbers of relay nodes for both the RLRS scheme and the RRS scheme. As shown in the figure, boosting the transmission power of source node leads to a noticeable reduction in the probability of transmission interruption across both schemes. This improvement is primarily owing to enhanced reception of relay nodes or intended destination, which strengthens overall communication link and reduces the risk of data loss or failure during transmission. More notably, under the RLRS scheme, the probability of transmission interruption further decreases with an increasing number of relay nodes. This result highlights the advantage of reinforcement learning in dynamically selecting the most suitable relay nodes for either forwarding the signal or assisting with cooperative jamming. By intelligently leveraging the spatial and channel diversity provided by a larger relay pool, the RLRS scheme effectively mitigates the risk of communication interruption, particularly in challenging or interference-prone environments. In contrast, the RRS scheme shows limited sensitivity to the number of relay nodes, as its selection process is random and does not account for channel state information or network conditions. Consequently, the benefits of increasing relay node density are not fully realized, and the probability of interruption remains relatively higher compared to the RLRS approach.

**Figure 5:**
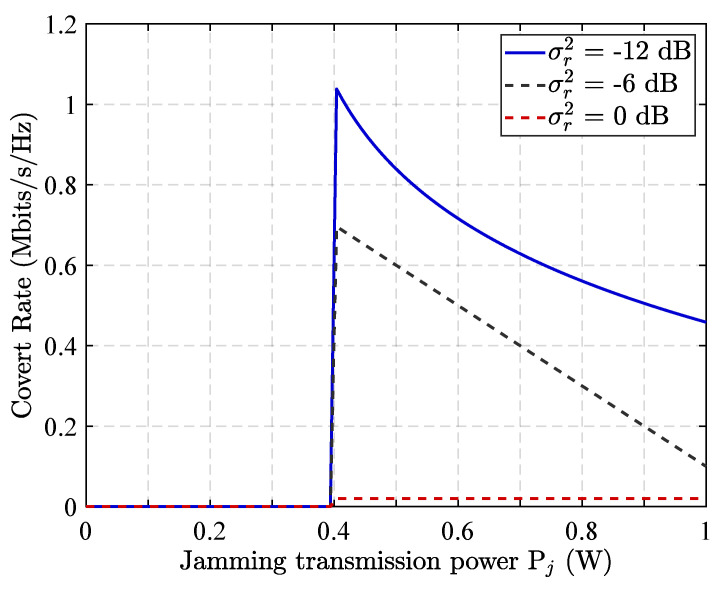
Impact of $P_{j}$ on covert rate under different numbers AWGN of relay in RLRS scheme.

The Figure 5 illustrates the impact of the interfering relay node’s transmission power on the communication concealment rate under various AWGN conditions. As shown, when the interfering node transmits at low power levels, the communication concealment rate remains near zero. This is because insufficient interference fails to effectively disrupt Willie’s detection capabilities, allowing accurate identification of covert transmissions. As the interference power increases, the concealment rate improves and eventually reaches a peak. However, further increases in interference power lead to a decline in the concealment rate. This degradation occurs because excessive interference begins to impair the forwarding of legitimate privacy signals, undermining the overall communication quality. Additionally, the intensity of AWGN at the relay node significantly influences the concealment performance. High levels of AWGN hinder the effectiveness of covert communication, making it difficult to maintain a satisfactory concealment rate.

**Figure 6:**
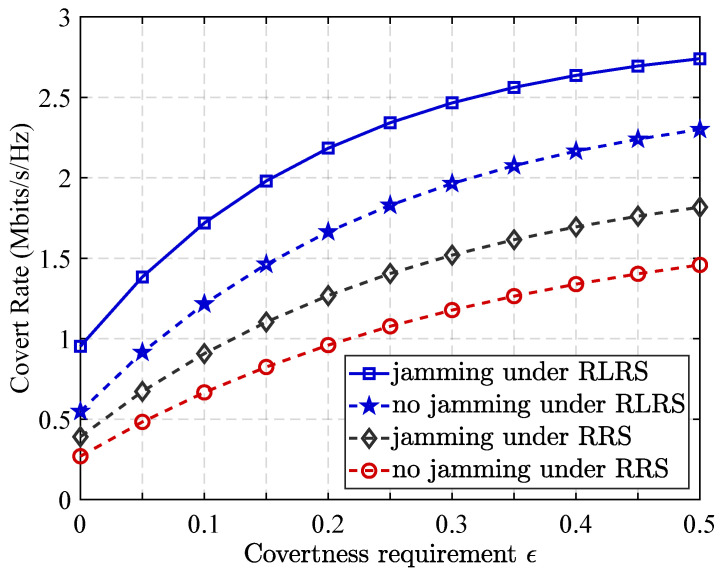
Impact of covertness requirement ϵ on covert rate under different relay selection scheme.

Figure 6 illustrates the impact of varying covert communication requirements on the system’s covert communication performance under different relay selection schemes. As illustrated in Figure 6, the achievable covert communication rate increases with the covertness parameter. This trend is because when ϵ is small, the covert requirement is more stringent (ξ≥1−ϵ close to one), which severely limits the transmit power and reduces the achievable covert rate. Conversely, when ϵ becomes larger, the constraint is relaxed, allowing higher transmit power and resulting in a higher covert rate. Furthermore, the results show that the RLRS strategy consistently outperforms the RRS scheme, and that cooperative jamming provides additional performance gains. In addition, the figure reveals a significant performance gap between relay selection schemes that incorporate cooperative interference and those that do not. Specifically, schemes that leverage cooperative jamming or interference assistance achieve substantially higher covert communication rates. This improvement is primarily due to the role of intelligently selected cooperative interference nodes, which are capable of injecting well-targeted noise or interference into the warden’s (Willie’s) observation channel. By doing so, they degrade Willie’s detection capability, thereby increasing the probability of successful covert transmission without being exposed.

## 6. Discussion and Future Work

Our study demonstrates the significant potential of a reinforcement learning-based approach for enhancing covert communications. However, the transition from theoretical models to practical, real-world deployment presents several challenges and opportunities for future research. In this section, we discuss the primary limitations of our current work and outline promising directions to address them.

Our simulation results validate the effectiveness of the proposed RLRS scheme. However, translating these theoretical findings into practical applications requires addressing several key challenges related to scalability, real-world implementation, and computational cost.

### 6.1. Real-World Channel Complexity and Model Adaptability

Our simulations rely on a standard wireless channel model using Rayleigh fading and additive white Gaussian noise (AWGN). While this approach is crucial for establishing a foundational understanding of the RLRS scheme’s performance, real-world channels are significantly more complex. Urban and industrial environments, key areas for IoT and military applications, often feature non-Gaussian impulsive noise, path shadowing, and Rician fading due to line-of-sight components.

To bridge this gap, future work should focus on training the RL agent on more realistic and diverse channel data. A promising solution is to employ transfer learning, where a model pre-trained in a simulated environment can be rapidly fine-tuned to adapt to the specific channel characteristics of a real-world deployment with a much smaller dataset, enhancing its practical applicability.

### 6.2. Computational Complexity for Resource-Constrained Devices

The MADDPG algorithm, while effective, is computationally intensive due to the training of multiple deep neural networks for the actor and critic functions. This poses a challenge for deployment on resource-constrained devices like IoT sensors, which have limited processing power and battery life.

A practical implementation strategy would involve a hybrid approach: the computationally heavy training phase can be offloaded to a powerful central entity (e.g., a base station or cloud server). Once trained, the resulting lightweight policy—the actor network—can be deployed onto the individual relays or sensors. Execution, which only requires a forward pass through this network to select an action, is far less demanding. For future research, exploring simpler RL algorithms or applying model compression techniques such as quantization and pruning could further reduce the computational footprint, making the system viable for a wider range of edge devices.

### 6.3. Adaptability to Dynamic Adversarial Strategies

Our current model assumes the colluding wardens employ a consistent detection strategy based on a radiometer with Equal Gain Combining (EGC). However, a sophisticated adversary may dynamically change its detection methods to counter our covert scheme. If a warden introduces a novel strategy not encountered during the agent’s training, the effectiveness of the learned policy could diminish.

To enhance robustness against such dynamic threats, the RL framework could be augmented with online or continual learning capabilities. This would enable the agents to continuously update their policies based on real-time feedback (e.g., successful transmission acknowledgments), allowing them to adapt to the warden’s evolving tactics. A more advanced direction would be to model the entire scenario as a dynamic game where both the communication system and the warden are intelligent, learning agents, leading to a more resilient co-adaptive strategy.

### 6.4. Resilience Against Insider Threats

The security of our proposed cooperative framework fundamentally relies on the assumption that all potential relays are trustworthy. The system is currently vulnerable to an insider threat, where one or more relays are malicious spies colluding with the wardens. Such a compromised relay could intentionally degrade performance by dropping packets, feeding false channel information to the RL agent, or remaining silent instead of jamming.

To mitigate this critical vulnerability, future work should integrate a trust management system. Such a system would monitor the performance and behavior of each relay over time. Relays that consistently underperform or whose actions correlate with failed transmissions could be assigned a decaying trust score. By dynamically excluding low-trust nodes from the pool of selectable relays, the system can build resilience against internal adversaries and ensure the integrity of the cooperative jamming and relaying strategy.

### 6.5. Scalability to Multi-User Scenarios

The system model presented in this paper focuses on a single-source, single-destination communication pair to provide a clear and foundational analysis of the RL-based relay and jammer selection mechanism. While this is a crucial first step, real-world networks often involve multiple sources and destinations operating concurrently. Extending our framework to such multi-user scenarios is a valuable direction for future research. This would introduce new challenges, including managing inter-user interference and designing a more complex multi-agent RL environment where agents must coordinate not only for covertness but also for spatial reuse of resources.

### 6.6. Limitations of Simulation and Path to Physical Implementation

We acknowledge that this study is based entirely on numerical simulations. This approach is essential for validating the algorithm’s theoretical performance under controlled and repeatable channel conditions. However, a gap invariably exists between simulated models and the complex, unpredictable nature of real-world wireless environments. The problem of maintaining covertness against sophisticated adversaries is highly relevant in practical fields like military communications and industrial IoT, where transmission detection can have severe consequences. Therefore, a crucial next step for future work is to validate our findings through physical experiments. Implementing the RLRS algorithm on a software-defined radio (SDR) testbed would allow for performance evaluation under real channel fading, hardware impairments, and interference, providing the ultimate proof of its practical viability.

### 6.7. Computational Complexity Analysis

A practical concern for any learning-based system is its computational cost, especially for deployment on resource-constrained devices like sensors or relays. The baseline RRS scheme has negligible computational complexity, as it involves a simple random selection. In contrast, our proposed RLRS scheme, based on the MADDPG algorithm, introduces a significant computational load, primarily during the training phase. This phase involves iterative updates to multiple deep neural networks (actor and critic), which requires substantial processing power and time.

However, the complexity during the execution phase is dramatically lower. Once the policy is trained, a relay only needs to perform a single forward pass through its lightweight actor network to select an action, which is a fast and efficient operation. To make the system practical, we propose a hybrid model where the intensive training is performed offline on a powerful central controller (e.g., a base station or cloud server). The finalized, trained policies are then distributed to the relays for low-latency, decentralized execution. This approach balances the need for intelligent adaptation with the operational constraints of network edge devices.

## 7. Conclusions

We investigate the problem of covert communications in multi-relay-assisted two-hop wireless networks under colluding warden scenarios and propose a RLRS scheme integrating cooperative jamming. By dynamically selecting optimal relays for signal forwarding and cooperative jamming nodes to emit interference, the developed framework optimizes the covert transmission rate under the constraint of maintaining Willie’s detection error probability above threshold. Through the design of a Markov game model and the application of the MADDPG algorithm, the system adaptively optimizes relay and jammer selection strategies based on network states. Simulation results demonstrate that contrasted with RRS approach, RLRS method significantly improves covert rate, reduces transmission outage probability, and enhances robustness against varying network parameters. These findings validate the effectiveness of reinforcement learning in dynamically optimizing relay and jammer selection for covert communications.

While our simulations validate the effectiveness of the RLRS scheme under standard Rayleigh fading and AWGN channels, a valuable direction for future research is to extend this framework to more realistic and complex communication environments. Future work should investigate the performance of our algorithm under non-Gaussian noise models, which are more representative of urban and industrial settings, and under alternative fading models such as Rician or Nakagami-m fading to account for different propagation scenarios. Testing the robustness of the RL agent in these more challenging, real-world conditions would be a crucial step toward practical deployment.

## Figures and Tables

**Table 1:** Simulation parameters.

Simulation Parameter	Value
Maximum transmit power for the source and jammers $P_{s}^{m}$ $P_{j}^{m}$ (dBm)	20
Noise power spectral density $N_{02}$	−174 dBm/Hz
Channel bandwidth *B*	1 MHz
Path loss exponent α	3.0
RL discount factor γ	0.99
MADDPG mini-batch size τ	128
Actor network soft update rate $\lambda_{a}$	0.001
Critic network soft update rate $\lambda_{b}$	0.001
Covertness requirement threshold ϵ	0.1

## Data Availability

Data are contained within the article.
